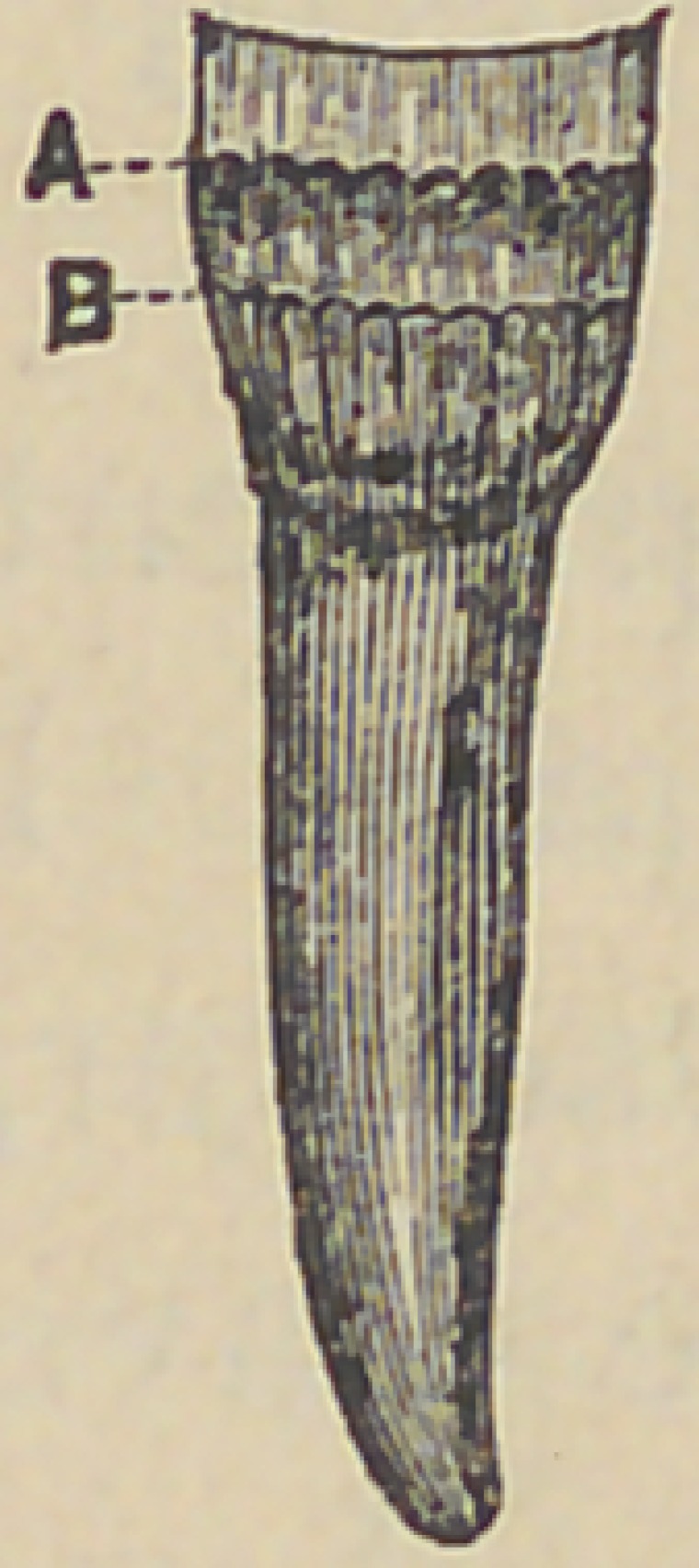# Transactions of the Mississippi Dental Association

**Published:** 1877-04

**Authors:** 


### TRANSACTIONS OF THE MISSISSIPPI VALLEY DENTAL ASSOCIATION

### DISCUSSIONS.

*Wednesday, March 7th, 1877.*

The first subject announced for discussion was, Abrasion of the Teeth, Chemical and Mechanical.

Discussion on first subject opened by Dr. J. Taft:

Abrasion is defined as a process of wearing or rubbing off. As it applies to the teeth, it may be either mechanical or chemical; in the former there is no elementary change of substance; the latter always involves such a change. By either method, so far as it applies to the teeth, the loss of substance occurs without occasioning any change in that which is left. Mechanical abrasion is easily comprehended—no obscurity attaches to it, usually the body producing the attrition is palpable and subject to control. The teeth sometimes occlude so as to wear each other; in other instances the teeth are used severely and persistently upon hard foreign substances that wear them rapidly away.

Chemical abrasion is by no means so easily understood; the cause and the process are involved in great obscurity; indeed, scarcely anything definite is known about them. There is quite a variety of manifestations, at least so far as results are concerned, sometimes rapidly removing the enamel over a large surface, and ceasing at the periphery of the dentine. In other cases transverse grooves are found upon the anterior superior teeth; and sometimes upon the posterior teeth as well. Sometimes it removes the enamel almost or quite entirely from the crowns of the teeth. In some cases it occurs only on the ends of the anterior teeth, and upon the masticating surfaces of the molars; it very rarely attacks the bicuspids on the masticating surfaces. On the molars the process usually forms cuppings, varying much in depth and diameter, in some instances it penetrates to the pulp chamber. Deep pits of small diameter are often presented, and in other cases the whole grinding surface will be cupped, having only a wall of enamel standing round the circumference. The inner surface of these cuppings are always as smooth and polished as perfect enamel.

This affection manifests itself in other ways than those I have referred to, but these are the most common. Now we shall be pleased to hear something of the actor and its mode of operation in this work.

Dr. Cassidy:—The softening of a tooth near the free margin of the gums, as described by Dr. Hunter, is, in my opinion, occasioned mostly by acetous fermentation; this is especially the case with the young between the ages of ten and fifteen years. The circumstances for the development of this process are more favorable than for any analogous series of acid forming reactions capable of producing the known results.

In the first place there is usually a greenish deposit on the part, (a vegetation, almost identical with the *mycodermi aceti*), which acts in the double capacity of affording a surface suitable for a quasi mechanical adhesion of starchy substances, and as an absorbent of oxygen. There assuredly follows fermentation. First: Change of starch into glucose. Second: Decomposition of the latter into alcohol and carbonic acid, similar to the juices of an apple passing into cider. Third: The breaking up of the alcohol into ethyle, aldehyde and water. Fourth: Aldehyde absorbing oxygen and thus forming acetic acid.

The acetates of the alkalies are soluble; the acetates of organic radicals are generally liquid.

Obviously the only preventive is the removal of the vegetation, and thereafter absolute cleanliness; and when filling is required, the continued application of a soft brush in connection with a mild alkali.

Dr. McCullom spoke of abrasion caused by the friction of the tooth brush. The snuff dippers, who brush their gums with soap and snuff, produce, by the persistent use of the brush, a number of parallel grooves on the enamel such as would be produced by the use of an emery tape. He has seen a woman brush her gums for an hour at one time. There are frequently grooves answering the above description, which must be ascribed to some constitutional weakness at the time the enamel was hardening. The effects of an attack of typhoid fever or flux is thus discernible years afterwards, especially if the disease was of long duration.

Dr. Rawls indorsed the last statement of Dr. McCullom. A softening of the substance of the tooth renders it more susceptible to the action of an abrading agent, which thus produces ravages in one case more readily than in another, where the conditions are apparently similar. He believes in the chemico-vital theory. The cases which present, where the teeth are worn down in cup-shaped depressions and exhibit a hard, smooth surface, indicate a disturbance of the nutritive matter which supplies the tooth. The chemical action which has taken place is no doubt the result of acids, but how do we account for the hardened surface of the abraded dentine? It is caused by the amœboid movements of the molecules in the disintegrated portions of the worn dentine wearing upon the floor of the cavity, abrading it until a point is reached where vital resistance checks it. He accounts for the susceptibility of a certain portion of the tooth, to the abrading influences on the hypothesis, that there are anastomoses of the dental tubuli which are restricted within certain planes, as illustrated in the diagram here given of a bicuspid tooth.

When the action has, (by comparatively slow stages), penetrated through the surface of the dentine immediately underlying the enamel, the *loops* of the tubuli being obliterated, their function is interfered with, and vital action ceases above the line or *plane* indicated at A. Below that line the *loops* being as yet unbroken, vital action continues unimpaired; so that when the destructive process has wasted all the dead tissue above, and reaches the still healthy structure below, it meets with a more marked resistance, and is often for a considerable time held in check, so as to present the vitrified surface which we often observe. But there is still a liability of a repetition of the process until finally the pulp is reached.

Dr. McCullom said that the only remedy for the evil, so far as he is aware, is filling.

Dr. McKellops:—This is a matter which he has dwelt on with much interest for a long time. All the discussions he had ever listened to failed to reach any final conclusion in relation to it. Cited the case of a girl, sixteen years old; she had *thirty-five* fillings in back teeth. Her father spoke of her inordinate fondness for apples, and attributed the destructive action to the acid of the fruit. It is remarkable that the peculiar species of decay spoken of is more frequently found to attack the teeth of the strongest men, men of immense bones and muscles; men who have never seen a day of sickness. These cup-shaped depressions appear and the teeth wear down to a flat, smooth surface.

Another very difficult species of decay to control, occurs at the necks of these teeth. The enamel is almost invaribly solid and dense. It is peculiar to elderly people—the young are not so affected. Had a patient whose bicuspids were worn down to a wedge shape; enamel all over the surfaces, hard and polished. The nutritive supply in such cases is not cut off. That does not explain it. In another case, a German seventy years old, grooves about the necks of the teeth were cut out and polished as smooth and clean as it is possible to conceive. No *brush* ever did that. Has seen such grooves *inside*, on the lingual surface of the molars. In one case he cut out and filled thirty-five grooves of the above description.

Dr. Taylor:—I have made a special effort to be present and hear this subject discussed. Am utterly in the dark. I can only say that Dr. McKellops has been a close observer, and his observations confirm my own exactly. We have this form of abrasion in the very best constitutions, where there have been really no special diseases by which we can account for them. I am really in want of information. I have learned nothing yet of a satisfactory nature in reference to this matter. We say there is mechanical abrasion, chemical abrasion, etc., yet how do we know? I have never seen but two or three cases where I was satisfied that there was chemical action at all, and how long that action had lasted, I do not know. I remark in relation to this matter, that you will generally find the gums in a healthy condition. You scarcely ever find a tooth missing. Constitution strong and vigorous, every indication of perfect health, and yet here are these structures wasting away! I am glad to find that we can arrest it by filling. The *cups* are perfect and need no excavation generally to enable them to retain a plug. I had a case where sixteen teeth were wearing out in this way. It began by mechanical abrasion and that was followed by chemical abrasion. Such teeth must be built up.

Another question I want to ask: How do you account for *sutures* in teeth? Why is it that the enamel organ at certain periods of life should cease its work, while the dentine organ goes on with its work? The pulp continues to perform its function, but at the same time there are imperfect deposits of the enamel on the outside of the teeth. What causes this? Why should scarlet fever, for instance, arrest one function and not disturb another so near to it? I think we are all in the dark on this question. Are we satisfied that this takes place always just at the time when the enamel is being deposited? I am not satisfied of that. Very often the children are healthy and robust.

Dr. Smith:—As to the characteristics of abrasion, we observe it only in old people; persons of strong, vigorous constitutions, and large muscular well developed jaws. He cited the case of a gentleman sixty-five years old. Teeth sensitive. Destroyed the pulps in lieu of capping. Shall I attempt to cap all these teeth? There would be twenty-five operations to perform.

Dr, McKellops:—Would cap them if the patient's pocket would bear it. (Laughter.)

Dr. Smith:—Have seen some heroic practice in the way of capping. Would not care to be in the place of the patient. It is an enormous thing to attempt, where there is so much to be done. In this case I propose to destroy the pulps from time to time.

Dr. McKellops:—I should certainly cap the teeth.

Dr. Taylor:—Do you find any patients under the age of thirty or forty years affected by abrasion of the teeth?

Dr. McKellops:—More particularly after forty years.

Dr. Taylor:—I learned a lesson not long ago. Had a patient who was in need of an operation of the kind. I declined to operate for him because he had heart disease, and I thought he would not live long anyhow. Dr. Taft built up several teeth for him and made one hundred and fifty dollars. The patient was very much pleased indeed. He has since died. (Laughter.)

Dr. Taft:—The most active case of abrasion I ever saw was in the case of a man thirty years old. Originally his teeth were firm and closed squarely together. The abrasion began with the superior central incisors and ultimately reached the laterals, cuspids and bicuspids. The lower teeth were somewhat affected. There was an elliptical opening nearly one-fourth of an inch in width between the teeth. No sensitiveness. This process had been in continuance two years. In some such cases there seems to be a greater density of the surface dentine than within. We ought to determine the kinds of constitutions in which the phenomenon occurs, and by microscopic examinations note the changes in the enamel structure. If there is eburnation the microscope will discover it. Experiments should be made to discover chemical reactions upon teeth in the mouth. Ascertain what agents will produce about the same kind of results as these results which are produced in the mouth.

Filling will not always check chemical abrasion. I have performed operations on the molars and have known the process to go on, undermining the filling and leave the plug of gold perfect.

Dr. Jennings:—Was there any discoloration under the filling?

Dr. Taft:—No sir, not usually.

Dr. Taylor:—If the enamel border is thick do you find such results occurring?

Dr. Taft:—I will say that in those instances I suspect the enamel was all gone and the eburnated surface of the dentine so far resembled the enamel as to mislead the operator. The abrasions upon the labial surface would indicate that the enamel is susceptible to its influence.

Dr. Taylor:—Do you know of any chemical agent which will produce phenomena similar to the abrasion which occurs in the teeth?

Dr. Taft:—Both acetic and lactic acid will produce a similar result, destroying the dentine without leaving a residuum, especially if they are in their nascent condition. (Subject passed.)

WEDNESDAY AFTERNOON.

The subject, Dental Caries; Cause and Prevention, was announced for consideration.

Dr. Hunter spoke of the decay which is accompanied by the green stain, and leaves the dentine very sensitive. Would like to know what can be done to prevent it.

Dr. H. A. Smith:—The main reliance of the profession is filling. In a vast number of cases it does not meet the requirement. Filling teeth, as a prevention of caries, is I think as a rule over rated; I think the profession promise their patients too much, and the more ignorant the dentist is the more he seems inclined to promise. In the class of caries to which Dr. Hunter refers, unless the cause is removed, filling is of no avail. My observation for the last few years has led me to doubt somewhat the efficacy of mere filling for the prevention of caries.

A great many approximate fillings must be done over many times. I have kept some statistics and I think that forty per cent of the fillings I make are approximate. These are largely in bicuspids and molars. Two-thirds of the operations I make are in the mouths of women. These bicuspid fillings do not always prevent decay. If they are put in at a period ranging from fourteen to twenty years they have to be done over in a majority of cases. We promise too much and the dental profession in order to place themselves right before the public have got to do something further than to treat teeth mechanically. Unless we do something for the coming generation we will lose the confidence of the public.

Dr. Rawls:—The remarks of Dr. Smith are worthy of consideration. Any one who has been in practice ten years has seen this very thing. What are we going to do about it? Of course none of us expect to save the teeth for a life time, but we can save them for a certain length of time, dependent very much on the location of the decay, the structure of the tooth, the material used, the condition of the secretions and the constitution of the patient. There is no doubt that decay will sometimes take place under the best of fillings. And no matter how perfect they may appear after they are put in, decay will re-commence, and the reason is that the tubuli of the tooth—the canals of the dentine—contain animal fluids, and these at times undergo disintegration. Oxygen effects an entrance and we have the same condition of affairs as before the filling was inserted. We can not get the dentine so perfectly dry as to prevent the accumulation of moisture. It shows its presence in the changed color of the tooth. Of course I make allowance for the structure of the tooth, and the condition of the dentinal canals. The bicuspids are the most difficult of all the teeth to save, for various reasons.

We have no reason however to feel discouraged and retire from the field.

Dr. Smith:—I do not propose to stop filling teeth, but I think the chances are that we are not fulfilling our mission in this age. The public is allowed to labor under a misapprehension as to the saving efficacy of our efforts in their behalf.

Dr. Clayton:—I have lately made a practice of using a layer of tin foil or a tin cylinder next the walls of cavities. Find that the tin thus used in connection with gold will save the tooth better than gold alone.

Dr. Hunter:—The question is how shall we prevent the existence of caries, whether produced by acids, or electricity, or any other agents?

Dr. Watt:—I sometimes feel discouraged in view of the little progress we, as a profession, make on this subject. If we are to judge by the reported discussions of the last two years, we have retrograded. I am sure I need not tell this society that there has been backsliding in regard to galvanic action within the mouth. Even in the papers read on this subject, we find many of the plainest elementary principles of galvanic action totally ignored. We read of solids, such as dentine, being electrolyzed, and of liquids which are absolute non-conductors of electricity, being subjected to the same impossible process. And by some it has been forgotten that decomposition is in proportion to the quantity of electricity passing through the liquid electrolyzed, and that, other things being equal, the power of excited surfaces varies inversely as the square root of the distance between them.

If we include chemical abrasion, there are four recognized varieties of dental caries; and, as chemical action is definite in its nature, there can be only as many direct or exciting causes. You might as well talk about forming water by varying the elements, or the proportions of its constituents as to talk of producing all the kinds of caries by a common, or single agent; or of producing one variety by various agents.

I am talking of only the direct or exciting causes of caries. Many substances injure the teeth, and thus become predisposing causes of caries, which are totally unable to produce *directly* any of the results known as dental decay. I have heard physicians upbraided by dentists for prescribing mineral acids, etc., without antidotes to preserve the teeth; but caries is never directly caused by these medicines. The quack on your streets, with his little sponge and "cleansing fluid," will whiten the teeth of a dirty mouthed boy, and then sell dozens of his bottles to the astonished spectators. The active principle of his fluid is usually hydrochloric acid, by which the teeth may be literally dissolved, while it thus acts only as a predisposing, but not as an exciting, cause of caries. The agents producing decay act only in their nascent condition, and unite with tooth substance as fast as formed or liberated. Bear in mind that chemical agents manifest increased energy in the nascent state. With us the babe is weaker than the man; in chemistry the reverse is true.

The most common decay is caused by hydrochloric acid. This is sometimes colorless, sometimes brown. The lime salts seem to be dissolved out, while most of the gelatinous portions remains in the cavity. The tendency of this decay is greatly increased by the excessive use of common salt. "Salt is good," but Americans use too much of it. In addition to the excess on our meats, we use it lavishly with celery, radishes, onions, eggs, etc., as if watching for an excuse to take "a little more salt." The line of prevention is plain, in this variety.

The white decay is caused by nitric acid. The penetrating nature of this variety has led some to look outside of chemical action for its exciting cause; but the phenomena of this are, on the chemical theory, simpler than those of any of the others. Ammonia is often found in the fluids of the mouth, and may be often detected in the breath. It is one of the alkalies, and of course, imparts an alkaline reaction to the buccal fluids. When these are alkaline, many dentists seem to think the teeth are comparatively safe, but they could scarcely be more widely mistaken. Ammonia is composed of nitrogen and hydrogen; and when exposed to oxygen, both of its elements are oxydized. But the oxydation of nitrogen forms nitric acid. Now when we remember that atmospheric oxygen has free access to the mouth, and that the gas bubbles in the saliva are rich in oxygen, the oxydation of ammonia, if present, is easily explained, and thus we can account for the presence of nitric acid, in its nascent state, and the consequent white decay.

You can often detect ammonia by the breath of your patient. When you find a ropy condition of the buccal fluids, you may suspect its presence. If you see fresh white caries, you have found its tracks, and now for prevention.

This state is a step towards scurvy, and acids are indicated, pickles, lemons, acid fruits generally, vinegar and water with sugar, etc., and these only with the regular meals. The acetic (vinegar) is the best of the acids, and the cheapest, but the choice of the patient may be consulted with safety.

Black decay differs from the rest in other respects as well as in color. There is much less disintegration of tissue. The lime salts are not removed to the same extent as in other varieties, but the decayed portion, though not broken down, cuts easily with an instrument. The lime is still there, but not in the same combinations as in natural tooth substance. You will readily infer that the destroying agent does not form soluble compounds with the lime salts, as in the first described variety. The contrasts between these two are very worthy of notice. Hydrochloric acid (the destroying agent in the first variety) acting on the carbonate of lime, forms chloride of calcium, while, at the same time, it freely dissolves the bone phosphate. But sulphuric acid, (the exciting agent of black decay) acting on the carbonate, gives sulphate of lime, (plaster of paris) a salt almost insoluble, and this acid almost totally fails to either decompose or dissolve the phosphate.

How do we account for the black color? We have been reminded, in former years, that several metals are found among the components of the teeth; but the color can not be satisfactorily explained in this way. But most of you are familiar with the carbonizing powers of sulphuric acid. When a common cork is used to confine it, the oxygen and hydrogen of the wood are removed, and the carbon is left in the shape of charcoal. A similar result has been often noticed from its action on animal tissue. The black color is due to the fact that the organic matter of the tooth is changed to *animal charcoal.* The phenomena of black decay are caused only by sulphuric acid in its nascent condition.

Persons with this decay in an active stage have, usually, the breath of a rotten egg, which is due to the presence of sulphuretted hydrogen, either free or combined with ammonia. When exposed to oxygen, both elements of sulphuretted hydrogen are oxydized, by which water and sulphuric acid are formed. Sulphuretted hydrogen is quite soluble, and if in the breath, will be dissolved in the fluids of the mouth, where its elements have all the facilities for oxydation, and the sulphuric acid thus formed, is, in its nascent state, in contact with the teeth.

In the black decay there is less breaking down of structure than in the other varieties, and accordingly it progresses less rapidly.

The tendency to this form of decay may be lessened by judicious dieting. Eggs, lean meats, onions, etc., are rich in sulphur, and should be avoided for a time. Bread, milk, butter, and fruits are better articles of diet for patients having this tendency. The vegetable acids have also a good effect.

A fourth variety, "chemical abrasion," has not been generally called caries by the text books; but the term applies to this as well as to the others. Please bear in mind that I am not at all talking about predisposing causes, but *about the thing that does the mischief.* A man tells his boy to split wood—the old man is the predisposing, but the boy, with the ax, is the immediate or exciting cause. The agent here makes clean work, leaves no litter, eats everything so far as it goes, leaving a smooth surface. To accomplish this, the agent must be able to dissolve the earthy and animal portions of the tooth with about equal facility. This complete removal of the destroyed tissue renders the investigation of this variety all the more difficult. But two acids seem to be capable of producing the phenomena, lactic and acetic, and these are sometimes found in the buccal fluids. In persons affected with rheumatism or gout, elderly persons with copious and high colored urine, you may certainly infer that the abrasion is due to lactic acid. Such persons should avoid, or use sparingly, milk, cheese, sour krout, and such vegetables as digest slowly. The grooves about the necks of the teeth of younger patients are probably oftener due to acetic than to lactic acid.

Dr. Taft:—I believe that Dr. Watt did not state the fact, but I presume that the lactic and acetic acids act only in their nascent conditions. Is not that the case?

Dr. Watt:—These acids like the muriatic acid mouth washes, will not cause caries, although by roughening the surface of the teeth they predispose to caries, but they operate to produce caries only in their nascent condition.

Dr. Taft:—The destruction which occurs to the teeth during a series of years is not always the work of one agent. I apprehend that two agents are frequently in the mouth at the same time, and that different agents may be operating upon the teeth at different times, from which fact I infer that we are in danger of confounding the phenomena presented by these agents, owing to the complications which result from the circumstance I have mentioned.

Dr. Watt:—A man forty years of age presented himself with a tooth one-third wasted away by chemical abrasion. White decay had set in, in the same cavity; in another tooth on the other side there was a cavity which had been produced by the black decay, in which the white decay had supervened.

Dr. Smith:—Do we ever have two agents operating at one and the same time?

Dr. Taft:—It is possible for two agents to operate at once in the mouth, but in different localities. I have more frequently found the hydrochloric acid decay in connection with the black decay than the white decay.

Dr. Leslie:—There has been little or nothing said in reference to predisposing causes of caries. I wish to speak of that matter. My theory is that caries proceeds largely from the interior of the tooth. For some reason the vital forces which built up that crystalline structure have become weakened, not entirely but to some extent. The element necessary for the maintenance of its individual atoms is lost. It is like the effort of a man who lifts a heavy weight—he raises it to a certain height and then his strength fails. So the vital force which holds the crystals of the tooth together is for some reason weakened. I have seen cases where a tooth was sensitive, a careful examination revealed no decay, yet in a year or two decay would appear at the point of sensitiveness. You frequently detect the presence of a cavity only by breaking through the crust of enamel, dark and discolored. That certainly indicates, according to my theory, a failure of the dental tubules to furnish pabulum. Afterwards the acids begin to operate. (Subject passed.)

## Figures and Tables

**Figure f1:**