# Erratum

**Published:** 1877-06

**Authors:** J. R. Clayton

**Affiliations:** Shelbyville, Ind.


### ERRATUM.

*Editor Dental Register:*

On page 162 of April No. of the Register, I am made by the reporter to say, that I made a practice of using a layer of tinfoil or cylinder against the walls of cavities. Find that the tin used in connection with gold will save the tooth better than gold alone

I said that I had used tinfoil in form of mat or cylinder against the cervical wall of bicuspids and molars, also the buccal walls of molars next the gum in certain conditions of the mouth.

So far, I have succeeded very well with gold and tinfoil so used, on grinding surfaces; no material answers the purpose as gold does.

J. R. CLAYTON,

Shelbyville, Ind.

April 27th, 1877.

